# Relationship between college students’ self-concept clarity and subjective well-being: the chain mediating roles of sense of control and meaning in life

**DOI:** 10.3389/fpsyg.2026.1772675

**Published:** 2026-05-21

**Authors:** Yinyin Dong, Min Xing, Huihui Wang, Yi Ai, Xinyao Dong

**Affiliations:** 1 The Faculty of Education, Henan Normal University, Xinxiang, China; 2 College of Education Sciences, Xinyang Normal University, Xinyang, China

**Keywords:** college students, meaning in life, self-concept clarity, sense of control, subjective well-being

## Abstract

**Objectives:**

Self-Determination Theory suggests that the human tendency toward integration is a fundamental prerequisite for self-actualization and the pursuit of well-being. This study extends this premise by investigating the relationship between self-concept clarity and subjective well-being, with a focus on the mediating underpinnings of sense of control and meaning in life. By modeling these variables through independent and chained mediation, we highlight the adaptive utility of the integrative self. Ultimately, this work maps the pathways to subjective well-being and informs the design of targeted educational interventions.

**Methods:**

A survey was conducted among 805 college students using the Self-Concept Clarity Scale, the Subjective Well-Being Scale, the Self of Control Scale, and the MIL judgments.

**Results:**

(1) self-concept clarity was significantly positively correlated with sense of control, meaning in life, and subjective well-being (*ps* < 0.05); sense of control was significantly positively correlated with meaning in life and subjective well-being (*p* < 0.05); meaning in life was significantly positively correlated with subjective well-being (*p* < 0.05). (2) The clarity of self-concept mainly affects subjective well-being through three pathways, namely: the mediating effect of sense of control (indirect effect = 0.04), the mediating effect of meaning in life (indirect effect = 0.08), and the chain mediating effect of sense of control and meaning in life (indirect effect = 0.02).

**Conclusion:**

The current study reveals that self-concept clarity exerts a significant influence on subjective well-being, a relationship primarily accounted for by the mediating roles of sense of control and meaning in life—particularly the role played by meaning in life. Consistent with Self-Determination Theory—which identifies the integrative tendency as a prerequisite for self-actualization and subjective well-being—these results highlight the adaptive utility of internal psychological structures. Beyond its theoretical contribution to the elucidation of well-being mechanisms, this work provides actionable insights for the implementation of targeted psychological support in higher education.

## 1 Introduction

Well-being, an enduring topic of human inquiry, has emerged as a central concern for both the government and the general populace. In recent years, Subjective well-being (SWB) remains a cornerstone of psychological inquiry, gaining increased urgency as recent data indicate a decline in well-being scores among college students—a demographic often characterized as the “academic elite” yet increasingly vulnerable to psychological distress (Hu et al., 2014; Jin et al., 2016; Xiang et al., 2021; Xue and Li, 2025). Within the modern university setting, students must navigate a complex landscape of intense academic pressure, evolving social support networks, and shifting cultural expectations. Consequently, it is vital to examine the mechanisms influencing SWB to develop effective, context-aware interventions. By investigating the “how” and “why” of well-being through the lens of adaptive motivation, we can move beyond theoretical definitions toward actionable pathways for student well-being programs. Previous research has found that life satisfaction is an important indicator of well-being, referring to an individual’s cognitive evaluation of his or her overall quality of life (Diener and Diener, 1995), which is manifested in subjective judgments regarding current living conditions, fulfillment of needs, and alignment with ideal life circumstances. The Self-Determination Theory postulates that as proactive organisms, all individuals have constructive, intrinsic, and innate integrative tendencies that lead people to strive to transform their social environments into internalized motives, an important prerequisite for self-actualization and the pursuit of well-being (Deci and Ryan, 1985). In the college context, this suggests that a student’s ability to thrive depends on how they navigate environmental stressors. Examining these intrinsic tendencies provides a blueprint for university mental health initiatives to foster resilience and self-actualization. Therefore, examining the adaptive functions of these intrinsic integrative tendencies holds significant value for both comprehending the nature of well-being and mapping effective pathways for its cultivation. This dual focus advances theoretical understanding while providing actionable insights for collective and individual well-being enhancement.

### 1.1 Self-concept clarity and subjective well being

Self-concept constitutes an individual’s perception of self-existence, and self-concept clarity is an important aspect of the structure of self-concept, referring to the degree of clarity of an individual’s understanding of the self, characterized by clarity, consistency, and stability (Campbell et al., 1996). In the realistic college environment, SCC acts as a psychological buffer; while students face identity shifts and academic challenges, those with high SCC maintain emotional equilibrium. Empirical evidence links high SCC to increased life purpose and decreased anxiety and suicidal ideation (Coutts et al., 2023). Empirical evidence demonstrates that self-concept clarity exhibits positive correlations with self-esteem, life purpose, emotional equilibrium, while showing inverse associations with depression, anxiety, loneliness, perceived stress, life adversity, and suicidal ideation/behaviors (Bigler et al., 2001; Coutts et al., 2023; Weber et al., 2023; Wong et al., 2019). Furthermore, SCC is a strong predictor of SWB, as it enhances a student’s adaptive capacity to handle academic demands and social adjustments (Nie and Gan, 2017; Xiang et al., 2021; Xiang et al., 2022). This psychological attribute signifies enhanced adaptive capacity (Bigler et al., 2001; Xu, 2007), enabling better social adjustment and performance in academic/professional domains, thereby corresponding with elevated subjective well-being. Based on these findings, the following hypothesis is proposed:

*H1*: College students’ self-concept clarity positively predicts their subjective well-being.

### 1.2 The mediating role of sense of control

Sense of control, or perceived control, refers to an individual’s belief in their capacity to govern internal states and behaviors, influence their environment, and achieve desired outcomes (Wallston et al., 1987). Empirical studies reveal that sense of control is positively correlated with health status, well-being, and subjective well-being, while inversely associating with depression, anxiety, and loneliness (Alonso-Ferres et al., 2020; Brailovskaia and Margraf, 2021; Gerstorf et al., 2014; Hong et al., 2021; Lachman and Weaver, 1998). For college students, a high sense of control is essential for mitigating the stress of high-stakes testing and career uncertainty. Research indicates that a sense of control facilitates effective problem-solving (Hong et al., 2021) and facilitates the alleviation of an individual’s emotional and physical responses to stress, including irritability, anxiety, meaninglessness, and despair (Neupert et al., 2007). Individuals with heightened sense of control demonstrate greater self-awareness and self-regulatory capacity, enabling them to better manage thoughts and behaviors, reconcile discrepancies between aspirations and reality, and employ effective problem-solving strategies. Jiang et al. (2022) found that individuals with low self-concept clarity exhibit diminished sense of control compared to their high-clarity counterparts. A clear, confident, and consistent self-concept promotes effective self-regulation and self-governance (Light, 2017). Because individuals with low SCC often report a diminished sense of agency, we suggest that SCC provides the stable foundation necessary for students to feel in control of their academic and personal lives. Consequently, we propose Hypothesis H2:

*H2*: Sense of control mediates the relationship between self-concept clarity and subjective well-being.

### 1.3 The mediating role of meaning in life

Meaning in life (MIL) denotes an individual’s perception that their existence is important, purposeful, and coherent (De Klerk, 2023), reflecting an awareness of value and purpose in one’s being (Steger et al., 2006). As a symbolic manifestation of human existence, MIL motivates individuals to establish goals, take action, and realize personal values, serving as both an intrinsic driver for well-being pursuit and a behavioral blueprint for its attainment, with the ultimate outcome being pleasure (life satisfaction). In a cultural context that often equates success with academic achievement, fostering a broader sense of MIL is a critical actionable outcome for student counseling. Seligman posits that pursuing meaning—even at personal cost or risk—constitutes a pathway to fulfillment; Diener et al. (2010) and Ryff and Singer (2008) equate meaningfulness with purposeful living, framing well-being as goal-directed existence (Seligman, 2012). Previous studies have found that MIL is closely related to individual well-being (Yang et al., 2016). Higher MIL correlates with enhanced mental health and well-being (Chen et al., 2022; Shen and Jiang, 2013), while demonstrating strong association with subjective well-being (Edwards and Van Tongeren, 2020; Steger et al., 2006; Shen and Jiang, 2013; Shen and Zhang, 2020), life satisfaction, and positive affect (Arslan and Allen, 2022; Jin et al., 2016). Since SCC allows individuals to align their actions with their values, it serves as a prerequisite for perceiving life as meaningful. It has been shown that self-concept clarity positively predicts MIL (Błażek and Besta, 2012; Nie and Gan, 2017; Shin et al., 2016), suggesting that individuals with a high level of self-concept clarity are more inclined to perceive their lives as meaningful. Thus, the following hypothesis is proposed:

*H3*: Meaning in life mediates the relationship between self-concept clarity and subjective well-being.

### 1.4 A chain mediation model: from theory to intervention

According to SDT, well-being is not merely the absence of stress but the result of proactive self-actualization. This process requires both a clear self-understanding and a perceived mastery over one’s environment. Recent studies suggest that sense of control and MIL are deeply intertwined; a student’s sense of agency often fuels their perception of purpose, and vice versa According to self-determination theory, as proactive beings, we are intrinsically motivated to achieve self-actualization and pursue well-being. This intrinsic motivation stems from an individual’s sense of self-awareness and self-control, driving people to strive to transform their social environment into a resource that serves as a source of subjective well-being (Arslan and Allen, 2022; Deci and Ryan, 1985; Gerstorf et al., 2014).

MIL plays a significant role in the relationship between reality and subjective well-being, but this subjective perception requires both a clear understanding of reality and the self, as well as a sense of control over one’s life and destiny (Edwards and Van Tongeren, 2020; Feng et al., 2020). Sense of control and MIL are critical factors influencing subjective well-being, both of which are closely related to the self-concept clarity. It has been shown that sense of control is significantly and positively correlated with adolescents’ meaning in life (meaning of existence or seeking meaning) (Deng and Kong, 2021; Hamama and Hamama-Raz, 2021; Li et al., 2019; Rasheed et al., 2023). Meaning in life has been shown to positively predict sense of control (Liu et al., 2022), alleviate anxiety by enhancing sense of control, and thereby improve the well-being of pre-retirement individuals (Miao et al., 2018). Conversely, sense of control positively predicts meaning in life, with heightened sense of control contributing to stronger perceptions of life’s purpose (Feng et al., 2020; Liu et al., 2022). By examining these constructs as a chain of mediation, this study identifies specific psychological levers for intervention. For example, university programs that bolster self-concept clarity may naturally enhance a student’s sense of control, subsequently deepening their sense of meaning and overall life satisfaction. Based on these findings, Hypothesis H4 is proposed:

*H4:* Sense of control and MIL act as chain mediators between self-concept clarity and subjective well-being.

**Figure 1:**
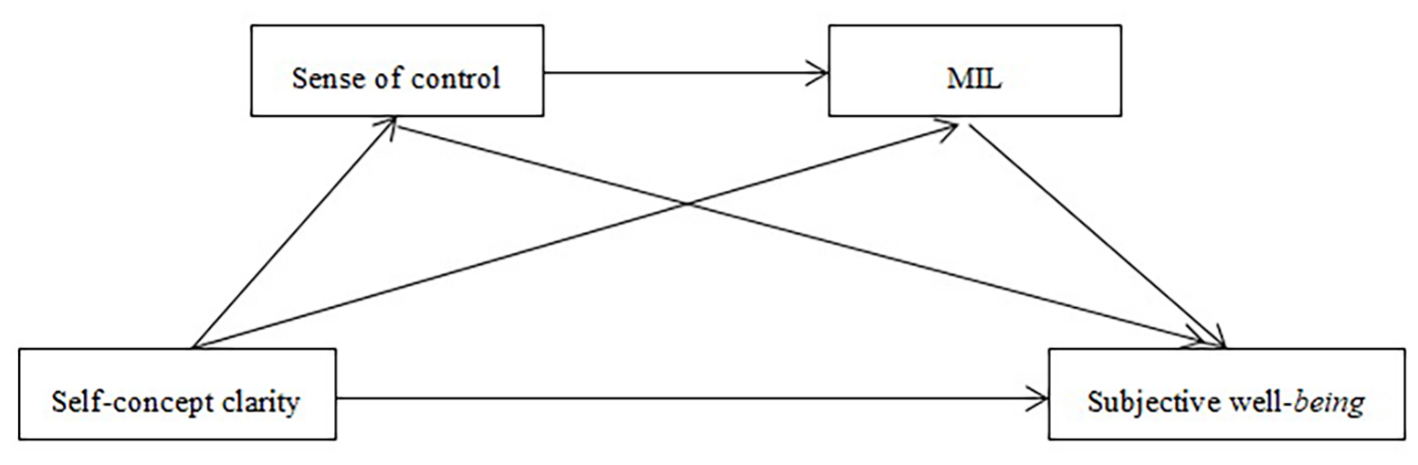
Theoretical model diagram.

In summarize, according to the self-determination theory, it is of great theoretical and practical significance to explore the adaptive motivation of “well-being” and the path to it. This study explores the mechanisms through which self-concept clarity influences the SWB of college students. By clarifying these adaptive motivations and constructing actionable pathways, we aim to provide educators and mental health professionals with evidence-based strategies to enhance student well-being amidst the pressures of contemporary campus life. The theoretical model is shown in Figure 1.

## 2 Method

### 2.1 Participants

To better determine an appropriate sample size, this study conducted a prior power analysis using G*Power 3.1. By setting the effect size f^2^ = 0.15, the significance level *α* = 0.05, and power (1-*β*) = 0.95, the sample size required for the chain mediation model—which includes four predictor variables (self-concept clarity, sense of control, MIL, and covariates)—was calculated to be 119.

Using the method of convenience sampling, 850 college students from two normal universities in Henan Province were selected for questionnaire survey, 837 questionnaires were recovered, and 805 valid questionnaires were obtained after collation due to incomplete answers and systematic operation (e.g., the questionnaire was only selected as 1), and the questionnaire validity rate was 96.2%. Among them, there were 231 male students (28.7%) and 574 female students (71.3%), aged 17–25 years old (Mage = 19.17, SD = 1.08). During the testing phase, participants completed the paper-and-pencil tests individually, and trained interviewers collect the questionnaires on-site to ensure that participants have answered all questions, unless they drop out midway. To control for common method bias, measures such as anonymous completion and explicit notification that there were no right or wrong answers were implemented, and the questionnaires were collected on-site. Post-hoc power analyses also confirmed that the final sample size (*n* = 805) provided sufficient power (1 − *β* = 0.99) to test the chain-mediation model.

This study was approved by the Research Ethics Committee of Xinyang Normal University (No. XFEC-2024-030). All participants voluntarily took part in the survey, and in accordance with the guidelines of the Declaration of Helsinki, each participant signed an informed consent form before beginning the survey. The survey took approximately 10 min to complete, and participants received a small gift as a token of appreciation upon completion.

### 2.2 Measurements

#### 2.2.1 Self-Concept Clarity Scale

The Self-Concept Clarity Scale developed by Campbell et al. (1996), translated by Niu et al. (2016), was used to measure the clarity and consistency of an individual’s self-concept. The scale consists of 12 items, such as “Some of my perceptions of myself are often in conflict with each other”, and is scored on a 5-point Likert scale ranging from 1 (“strongly disagree”) to 5 (“strongly agree”), with higher scores indicating greater self-concept clarity. In this study, the alpha coefficient of the scale was 0.680. Further analysis shows that McDonald’s *ω* is 0.723, which meets the basic requirements for further data analysis.

#### 2.2.2 Subjective Well-Being Scale

According to previous studies, the Life Satisfaction Questionnaire developed by Diener et al. (1985) was used to measure individuals’ subjective well-being (Niu et al., 2015), which has five items, such as “I am satisfied with my life”. Participants rated their agreement with statements such as “I am satisfied with my life” on a 7-point Likert scale (1 = strongly disagree to 7 = strongly agree), with higher total scores reflecting greater life satisfaction. As one of the most widely used instruments for assessing life satisfaction, the reliability coefficient alpha of the questionnaire was 0.723 in this study.

#### 2.2.3 Self of Control Scale

Sense of control was assessed using the 5-item scale developed by Costin and Vignoles (2020), which is grounded in compensatory control literature and operationalized based on validated manipulations (Kay et al., 2008). The scale consists of five items, such as “The events in my life are mainly determined by my own actions”, and is scored on a 7-point Likert scale ranging from 1 (strongly disagree) to 7 (strongly agree). Higher scores indicate a greater sense of control. In this study, the alpha coefficient for this scale was 0.617, further analysis shows that McDonald’s ω is 0.609, which meets the basic requirements for further data analysis.

#### 2.2.4 MIL judgments

Costin and Vignoles (2020) measure of meaning in life was used. The scale consists of four subscales with a total of 16 items for measures of general meaning in life, such as “My life as a whole is meaningful,” and three components: coherence, such as “I can make sense of what is going on in my life”; purpose, as in “I have some goals in life that compel me to keep going”; and significance, as in “I can say that my life is significant even considering how big the universe is.” The scale is based on a 7-point Likert scale (1 = strongly disagree to 7 = strongly agree), with higher scores indicating stronger meaning in life. In this study, the alpha coefficients for the total scale and the four subscales were 0.851, 0.839, 0.642, 0.704, and 0.756, respectively.

### 2.3 Questionnaire validity

This study established a multi-tiered quality assurance mechanism covering three aspects—scale selection, test administration procedures, and data screening—to ensure the questionnaire validity. To ensure construct validity, confirmatory factor analysis (CFA) was conducted on each questionnaire using AMOS 20.0. The fit indices for each scale were acceptable: χ^2^/df < 3, IFI/TLI/CFI > 0.90, and RMSEA < 0.08. Additionally, average variance extracted (AVE) and composite reliability (CR) were calculated to assess convergent validity. The AVE values for all dimensions ranged from 0.50 to 0.64 (≥0.50), and the CR values ranged from 0.81 to 0.92 (≥0.70), confirming good convergent validity (Alonso-Ferres et al., 2020).

### 2.4 Data analysis

Descriptive statistics, correlation analysis were conducted using the Statistical Package for the Social Sciences (SPSS 22.0). Then, regression analysis and mediation effect analysis were performed using the PROCESS 3.2 plug-in for SPSS 22.0. A structural equation model was constructed using AMOS 20.0 software, with gender and age included as control variables. The Bootstrap method (5,000 resamples) was used to test for mediating effects. Model fit indices were considered acceptable χ^2^/df < 3, IFI, TLI, and CFI > 0.90, and RMSEA < 0.08. The significance level was set at *α* = 0.05.

The bias-corrected percentile Bootstrap method was adopted to analyze the mediating roles (Fang et al., 2012). Randomly select participants from 5,000 bootstrap resamples and use Model 6 in PROCESS to estimate the 95% Bootstrap confidence interval for the mediating role. The 95% confidence interval does not contain 0, indicating that a significant level was reached. Exploratory factor analysis was conducted on all the questions of the questionnaire using the Harman one-way test (Tang and Wen, 2020). The results showed that there were 11 factors with an eigenroot greater than 1. The variance explained by the largest factor was 18.27% (less than 40%); therefore, there was no serious common method bias in this study.

## 3 Results

### 3.1 Descriptive statistics and correlations

The means, standard deviations, and correlation coefficients of Self-concept clarity, sense of control, MIL, and subjective well-being of 805 college students are shown in Table 1, from which it can be seen that Self-concept clarity and sense of control (*r* = 0.25, *p* < 0.01), MIL (*r* = 0.34, *p* < 0.01), and subjective well-being (*r* = 0.22, *p* < 0.01) are all significant positive correlations; sense of control was significantly positively correlated with MIL (*r* = 0.47, *p* < 0.01) and subjective well-being (*r* = 0.26, *p* < 0.01); MIL was significantly positively correlated with subjective well-being (*r* = 0.27, *p* < 0.01).

**Table 1:** Means, standard deviations and correlations among the variables.

Variable	Means	Standard deviations	1	2	3	4
1. Self-concept clarity	34.51	5.87	—			
2. Sense of control	24.85	3.61	0.12^**^	—		
3. MIL	86.72	10.80	0.34^**^	0.47^**^	—	
4. Subjective well-being	20.00	4.86	0.22^**^	0.26^**^	0.27^**^	—

^**^p < 0.01.

### 3.2 Testing for chain mediation role

To examine the mechanisms underlying the relationship between self-concept clarity and subjective well-being, we conducted a mediation analysis while controlling for gender and age. As shown in Table 2, self-concept clarity was a significant predictor of subjective well-being, sense of control, and Meaning in Life (MIL). As displayed in Table 2, the total effect of self-concept clarity on subjective well-being was significant (*β* = 0.43, *p* < 0.001); and it significantly and positively predicted sense of control (*β* = 0.19, *p* < 0.001) and MIL (*β* = 0.41, *p* < 0.001). With the addition of sense of control and MIL, the predictive role of self-concept clarity on subjective well-being remained significant but was reduced (*β* = 0.29, *p* < 0.01). Sense of control significantly and positively predicted MIL (*β* = 0.40, *p* < 0.001) and subjective well-being (*β* = 0.23, *p* < 0.001). MIL significantly and positively predicted subjective well-being (*β* = 0.20, *p* < 0.001).

**Table 2:** Regression analysis among variables.

Regression equations		Overall fit indices			Significance of regression coefficients	
Predictor variables	Outcome variable	*R*	*R^2^*	*F*	*β*	t
Sex	Subjective well-being	0.22	0.05	13.10***	0.02	0.23
Age					0.02	0.83
Self-concept clarity					0.43	6.22***
Sex	Sense of control	0.14	0.02	5.04***	0.03	0.51
Age					0.03	1.61
Self-concept clarity					0.19	3.57***
Sex	MIL	0.56	0.31	91.48***	0.18	4.18***
Age					−0.02	−1.32
Self-concept clarity					0.41	9.97***
Sense of control					0.40	14.61***
Sex	Subjective well-being	0.34	0.11	20.66***	−0.03	−0.40
Age					0.02	0.63
Self-concept clarity					0.29	4.04***
Sense of control					0.23	4.60***
MIL					0.20	3.50***

^**^p < 0.01, ^***^p < 0.001.

**Figure 2:**
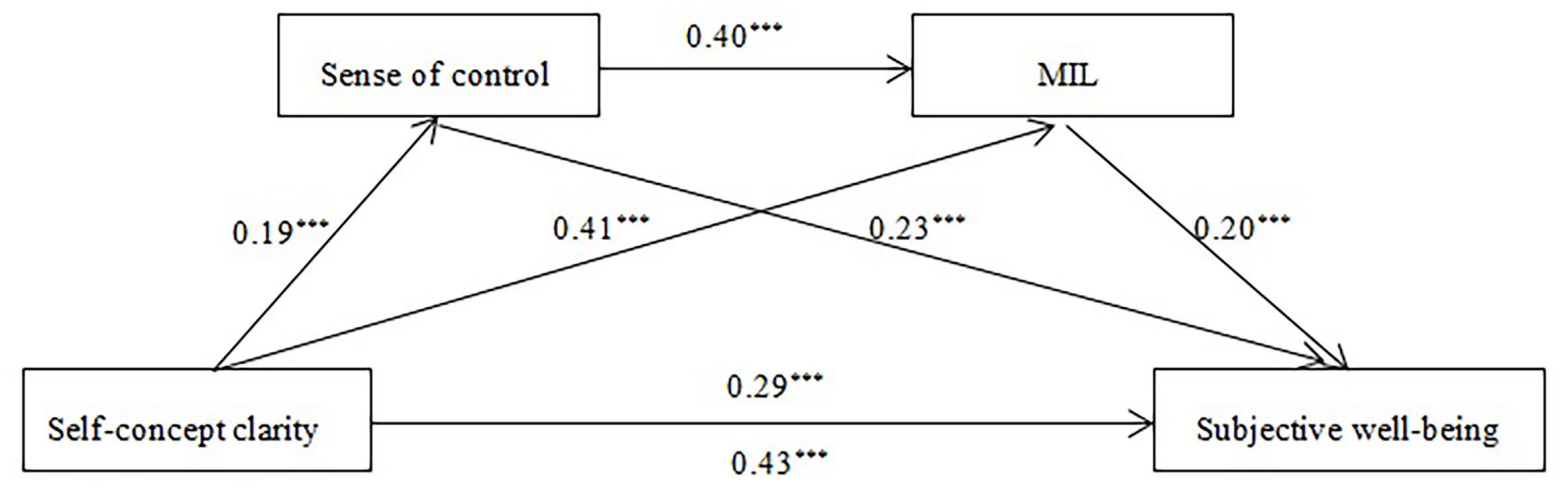
The chain mediating model (*N* = 805). ***p < 0.001.

As illustrated in Table 3 and Figure 2, the mediation results indicate that the relationship between self-concept clarity and well-being is partially explained by both sense of control and MIL. Notably, the pathway through Meaning in Life (MIL) emerged as the most substantively significant mediator, accounting for 19.31% of the total effect (indirect effect = 0.08, 95% CI [LLCI, ULCI]). While the serial mediation (Self-concept clarity → Sense of control → MIL → Well-being) and the independent sense of control pathway were also statistically significant, their relative contributions were smaller. The three indirect effects accounted for 10.14, 3.54, and 19.31% of the total effect (0.43), respectively. Bootstrap 95% confidence intervals for all three indirect effects excluded zero, confirming their statistical significance.

**Table 3:** Analysis of the mediating effect of sense of control and mil in the effect of self-concept clarity on subjective well-being.

Path type	Effect size	Boot SE	Boot CI lower limit	Boot CI limit	Proportion effect size
Total effect	0.14	0.04	0.08	0.21	32.99%
Indirect effect 1	0.04	0.02	0.02	0.08	10.14%
Indirect effect 2	0.02	0.01	0.01	0.03	3.54%
Indirect effect 3	0.08	0.03	0.04	0.14	19.31%

The prominence of the MIL pathway suggests that a clear self-concept does not merely improve well-being through a sense of agency (control), but more significantly by providing students with a coherent framework that serves as the fundamental prerequisite for self-actualization and the pursuit of well-being. Theoretically, this underscores the role of self-concept clarity as a prerequisite for existential meaning-making. Practically, these findings suggest that university mental health interventions should move beyond general self-esteem building; instead, they should focus on helping students clarify their self-identity to facilitate a deeper sense of meaning in life, which appears to be a primary driver of long-term subjective well-being.

## 4 Discussion

The present study focused on the adaptive motivation and accessible paths to “well-being.” The results show that self-concept clarity directly and significantly positively predict subjective well-being and indirectly affect subjective well-being through the individual mediating roles of sense of control and MIL. At the same time, it also indirectly affects subjective well-being through the chain mediating roles of sense of control and MIL.

### 4.1 The role of self-concept clarity on subjective well-being

Self-concept clarity (SCC) refers to the extent to which self-beliefs are clearly defined, confident, consistent, and stable. Our findings indicate that SCC refers to the extent to which self-beliefs are clearly defined, confident, consistent, and stable. Our findings indicate that SCC significantly predicts subjective well-being (SWB) among college students, aligning with established research (Błażek and Besta, 2012; Coutts et al., 2023; Nie and Gan, 2017; Xiang et al., 2021).

College students with high self-concept clarity maintain a stable and clear understanding of their personality traits, academic abilities, strengths, and personal needs (Xu, 2007). Furthermore, research indicates that self-concept clarity serves as a significant buffer against stress. From a cognitive perspective, high SCC functions as a “self-anchor” that reduces internal ambiguity. Individuals with a clear self-understanding are better equipped to identify their intrinsic needs, facilitating the pursuit of congruent goals and enhancing life satisfaction (Coutts et al., 2023; Yang et al., 2016). Conversely, low SCC often results in identity confusion and heightened susceptibility to environmental stressors, which diminishes well-being (Zhu, 2010). Thus, SCC serves as a critical psychological resource that provides the cognitive stability necessary for emotional flourishing. Therefore, improving self-concept clarity among college students may strengthen stress resilience, mitigate negative emotions (e.g., anxiety, depression), and ultimately elevate their subjective well-being.

### 4.2 The chain mediating role of sense of control and meaning in life

This study identifies a significant chain mediation role, where in SCC influences SWB through the sequential operation of sense of control and meaning in life (MIL). Viewed through the lens of Self-Determination Theory (SDT), this mechanism represents a transition from cognitive-emotional self-regulation to adaptive motivation (Deci and Ryan, 1985). Self-concept clarity, as the foundation of individual pursuit of “well-being,” facilitates individual reflection on the MIL in a holistic manner through the sense of control, and prompts the individual to carry out constructive and internal integration that transforms the social environment into adaptive motivations for well-being.

SCC provides the structural foundation for a robust sense of control. When individuals possess a clear self-schema, they perceive themselves as capable agents rather than subjects of external chance (Gang, 2022; Light, 2017; Smith et al., 1996; Rasheed et al., 2023). This sense of control functions as a core component of cognitive-emotional self-regulation, enabling students to manage unpredictable situations, resolve goal conflicts, and inhibit impulsive desires (Hofmann et al., 2014; Ou et al., 2016), which directly enhance subjective well-being (Zhang et al., 2022). Sense of control helps individuals construct a sense of meaning in life; individuals with low control have difficulty regulating bad emotions and are prone to anxiety, and frequent negative emotions increase the risk of anxiety and depression, which makes individuals less likely to experience the value and meaning of life (Weger and Sandi, 2018). By facilitating effective behavioral regulation, SoC reduces negative affect and fosters the agency required for sustained well-being (Hu et al., 2022; Wu et al., 2023).

In addition, self-concept clarity is important for experiencing meaning in life; when people understand themselves and their relationships with others, they can confirm the value of their self-existence and thus experience a sense of meaning in life. A coherent self-concept provides a stable framework for interpreting environmental interactions and integrating external experiences without identity confusion. This cognitive stability allows individuals to derive value from life narratives, forming the basis of meaning in life (Shin et al., 2016).

Notably, our model indicates that MIL exerts a stronger mediating role on SWB than sense of control. This finding has important implications for theoretical models of well-being: while sense of control is a functional/instrumental mediator—the “how” of self-regulation—MIL is a teleological/existential mediator that provides the “why.” MIL represents the ultimate psychological integration of the self-concept and the environment. Within the SDT framework, while control (linked to competence and autonomy) is essential, the internalization of meaning is what truly sustains long-term psychological health (Liu et al., 2022). While sense of control provides the capacity for regulation, MIL provides the purpose for it. Our results suggest that sense of control facilitates the construction of MIL; individuals who feel in control of their lives are better able to align their actions with long-term values, deriving a sense of coherence from their experiences (He and You, 2006; Hu et al., 2022). In this context, MIL serves as an adaptive motivational process—it transforms the social environment into internal kinetic energy, allowing individuals to face hardships with resilience and a sense of fulfillment (Hu et al., 2014; Liu et al., 2022).

In summary, the path from SCC to SWB operates through a sequential mechanism: a clear self-concept bolsters regulatory capacity (sense of control), which in turn fosters existential purpose (MIL), ultimately culminating in enhanced well-being. This suggests that mental health interventions should move beyond skill-building for environmental mastery and focus on helping students integrate their self-knowledge into a cohesive, meaningful life narrative (Hu et al., 2022; Wu et al., 2023). Based on existing research findings, the future development of experiential and reality-based self-awareness courses—which enhance students’ ability to integrate resources related to both their external environment and intrinsic motivation—is of great significance in helping college students improve their mental health and pursue well-being (Gao and Mohamad, 2025).

### 4.3 Limitations and future research

The study’s sample predominantly consisted of first-year college students. This pivotal transition from secondary to higher education often entails significant shifts in self-concept and perceived self-control, which may account for the diminished reliability observed in certain scales. Furthermore, the pronounced gender imbalance—characterized by a high concentration of female students within the teacher education program—presents a limitation that necessitates further inquiry. The homogeneity of the participant group may constrain the external validity of these findings. Future research should aim to enhance validity by expanding the sampling frame, increasing sample size, and ensuring a more balanced distribution across gender and grade levels. Finally, it should be noted that the data were collected via self-report measures, which may introduce subjective bias. While questionnaires were administered in centralized offline batches with professional guidance, and statistical analyses confirmed the absence of multicollinearity (covariance issues), the reliance on self-reported data remains susceptible to environmental influences and the participants’ transient emotional states. To enhance the objectivity of future findings, researchers should diversify data collection methods and integrate qualitative approaches, such as structured observations and interviews, to facilitate triangulation and cross-validation of results.

## 5 Conclusion

In the present study, self-concept clarity emerged as a direct, positive predictor of individuals’ subjective well-being. Furthermore, our findings demonstrated an indirect sequential pathway, suggesting that self-concept clarity is associated with a greater sense of control, which in turn relates to a stronger meaning in life (MIL) and ultimately higher subjective well-being. Most importantly, MIL appears to serve as an adaptive motivational factor on the path to well-being. By illustrating how self-concept clarity and sense of control may facilitate the integration of meaning in life, these associations lend further empirical support to Self-Determination Theory. Importantly, while these observed relationships highlight potential targets for interventions aimed at enhancing self-concept clarity, perceived control, and meaning in life, we maintain that these represent structural associations rather than definitive causal links.

## Data Availability

The raw data supporting the conclusions of this article will be made available by the authors, without undue reservation.
